# Effects of peanut shells serving as a carbon source and microbial carrier on bacterial community structure in *Penaeus vannamei* culture water

**DOI:** 10.3389/fmicb.2026.1778957

**Published:** 2026-04-10

**Authors:** Xiaoshuang Liu, Chengjia Wu, Ke Wang, Naresh Kumar Dewangan, Panpan Li, Xinping Yu, Guangqing Yu, Zhenjiang Yang, Zhunan Li, Pengsheng Dong

**Affiliations:** 1 College of Animal Science and Technology, Henan Agricultural University, Zhengzhou, China; 2 Shri Shankaracharya Professional University, Bhilai, India

**Keywords:** 16S rRNA gene sequencing, bacterial community succession, biofloc technology, gentler perturbation, peanut shells, *Penaeus vannamei*

## Abstract

Intensive shrimp aquaculture systems often suffer from water quality deterioration and disease outbreaks. Biofloc technology is one of the sustainable management strategies for shrimp culture, while the choice of organic carbon source critically influences microbial community dynamics. This study investigated the effects of peanut shells (an abundant agricultural by-product) on the bacterial community in shrimp rearing water in *Penaeus vannamei* culture systems. Over a 28-day controlled experiment, the peanut shell–amended system exhibited significantly lower ammonia and nitrite concentrations than the control, indicating improved nitrogen removal. Overall bacterial diversity and community structure showed only moderate shifts with no significant change compared with the control, the relative abundances of *Bacteroidetes*, *Flavobacteriaceae*, and *Saprospiraceae* showed increasing trends, but were not statistically significant. Redundancy analysis identified phosphate and nitrate as key environmental factors associated with community succession. Functional prediction showed that peanut shells enhanced pathways related to quorum sensing, secretion systems, and organic matter degradation, while reducing biosynthetic activity. Neutral community modeling further revealed that peanut shells did not notably alter the assembly processes across the whole culture period, and these limited changes were mainly attributed to the selective enrichment of potential beneficial bacteria such as *Rhodobacteraceae* taxa. These findings indicate that peanut shells exert a gentler perturbation on the microbial ecosystem compared to readily degradable carbon sources like molasses or glucose, which are known to provoke more drastic community reassembly. This work highlights the potential of peanut shells as a sustainable carbon supplement for improving water quality in a sustainable manner in shrimp aquaculture.

## 1 Introduction

*Penaeus vannamei* is currently the world’s most widely farmed shrimp species due to its tolerance of varying salinity and temperature, rapid growth, and high economic value. It provides an important source of animal protein for humans and holds a central position in global shrimp aquaculture (Sicuro, 2021). However, the sustainable development of this industry faces significant challenges. Intensive aquaculture systems are often associated with excessive feed input, resulting in the continuous accumulation of residual feed and excrement. This leads to a significant increase in the concentration of toxic nitrogenous compounds such as ammonia (NH₄^+^–N) and nitrite (NO₂^−^–N), which pose serious threats to shrimp physiological health, including inhibiting growth performance, and may even lead to high mortality (Crab et al., 2007; Wang et al., 2020). Moreover, the deteriorating water environment subjects shrimp to prolonged stress, leading to suppressed immunity and increased vulnerability to opportunistic pathogens such as *Vibrio* spp., thereby causing frequent disease outbreaks (Flegel, 2019). The acute and chronic toxic effects of ammonia and nitrite on *Penaeus vannamei* are well documented and threaten shrimp survival and growth (Lin and Chen, 2001; Romano and Zeng, 2013). Historically, the aquaculture industry has relied on antibiotics or chemical drugs for disease prevention and control, but this approach is becoming increasingly ineffective but also brings a series of problems, such as increased drug resistance of pathogens, drug residues, and environmental pollution (Lulijwa et al., 2020). Therefore, the development of efficient, environmentally friendly, and sustainable healthy aquaculture technology has become a major need to be solved urgently in the shrimp aquaculture industry.

The structure and function of microbial communities are fundamental to maintaining the health and stability of aquaculture systems. An ideal microbial community efficiently converts toxic nitrogenous compounds such as ammonia, nitrite, and nitrate through nitrification, denitrification, and related processes, thereby preserving ecological balance in the culture water. Beneficial microorganisms can also suppress the proliferation of pathogenic bacteria such as *Vibrio* through competitive exclusion and the secretion of antimicrobial substances (Yadav et al., 2025). This microecological strategy provides a promising pathway toward antibiotic-free aquaculture, making the targeted cultivation of beneficial microbial communities a key scientific objective. In this context, biofloc technology (BFT) provides a controllable approach for shaping functional microbial communities by regulating the type and dosage of exogenous carbon sources. BFT regulates aquaculture systems by shaping the assembly and activity of microbial communities, thereby enabling microecological regulation of water quality and system stability (Torres-Lagos et al., 2024). Previous studies have shown that the choice of carbon source directly influences microbial composition and ecological functions within the system. For example, glucose addition has been reported to improve shrimp health and aquaculture performance by promoting the assembly of specific bacterial groups such as *Rhodobacteraceae* in the culture system (Dong et al., 2023). Similarly, sucrose addition has been shown to improve shrimp health and system stability by enriching core taxa belonging to *Actinobacteria* and *Rhodobacteraceae* across water, biofloc, and shrimp gut (Guo et al., 2022), contributing to nitrogen transformation and preventing nitrate accumulation in the culture environment. Additional carbon sources may therefore jointly contribute to the establishment of a more stable and resilient aquaculture micro-ecosystem (Shi et al., 2022). Accordingly, carbon source management represents a key mechanism for regulating microbial community function and acts as a core link between BFT system structure and ecological outcomes. However, different types of carbon sources exhibit distinct physicochemical properties and degradation characteristics, which may lead to divergent microbial responses and regulatory outcomes in BFT systems.

In BFT systems, the physicochemical properties of carbon sources largely determine microbial community dynamics. Readily degradable carbon sources such as molasses, glucose, and starch can rapidly drive obvious differentiation in microbial community composition and structure within a short time frame (Dong et al., 2023; Guo et al., 2022; Zhang et al., 2024). However, their rapid intervention often causes sharp fluctuations in dissolved oxygen and pH, leading to pronounced disturbances in microbial community structure and composition (Crab et al., 2012; Ray et al., 2019). To mitigate such instability, lignocellulosic agricultural by-products have been proposed as alternative carbon sources. Due to their complex polymeric structure, these materials typically degrade more slowly and release carbon gradually, which may support sustained stability in microbial community composition and structure. Accordingly, several lignocellulosic agricultural residues have been explored in biofloc systems as alternative carbon sources. Through stimulating heterotrophic assimilation as well as nitrification and denitrification processes, BFT enhances the capacity of microbial communities to remove toxic nitrogenous compounds and maintain water quality (Khanjani et al., 2025). For example, sugarcane bagasse can enrich cellulose-degrading bacteria such as *Cellulomona*s and *Clostridium*, thereby enhancing solid waste decomposition and alleviating substrate accumulation (Chen et al., 2022; Wu et al., 2022). Rice bran has been reported to promote beneficial taxa, including *Flavobacterium* and *Bacillus*, which contribute to ammonia assimilation and pathogen suppression (Romano et al., 2018). Similarly, corn cob amendment supports denitrifying bacterial groups such as *Rhodocyclaceae*, facilitating nitrogen removal and improving water quality (Lan et al., 2023). Peanut shells are a widely available lignocellulosic agricultural by-product characterized by a high carbon–nitrogen ratio and a porous fibrous structure (Li et al., 2022; Manimekala et al., 2025). Similar to other lignocellulosic materials, peanut shells can serve as both carbon sources and microbial attachment substrates (Jiao et al., 2025). However, the ecological regulatory mechanisms of complex lignocellulosic substrates (such as peanut shells) in BFT systems remain largely unexplored compared with those of simple organic carbon sources (such as glucose and sucrose). Therefore, peanut shells were selected as one type of slow-degrading agricultural residue in this study to explore how gradual carbon inputs influence microbial community succession within BFT systems. By integrating aquaculture environmental parameters with microbial functional characteristics, we aimed to comprehensively evaluate the regulatory effects of peanut shells on system stability and nitrogen transformation processes. Based on these considerations, we hypothesized that the gradual degradation of peanut shells would exert a moderate and sustained influence on the bacterial community succession and functional profile, thereby avoiding the strong selective pressure typically associated with readily degradable carbon sources. This study therefore evaluates how peanut shell amendment shapes the taxonomic structure and functional potential of microbial communities in shrimp culture water, thereby providing insight into the ecological mechanisms of slow-release carbon regulation in BFT systems.

In summary, in this study, a 28-day culture experiment was conducted to examine how peanut shell amendment, acting as a slow-releasing carbon source and microbial attachment substrate, influences water physicochemical properties, bacterial taxonomic composition (from phylum to genus/OTU levels), bacterial community succession, assembly processes and predicted functional profiles in *Penaeus vannamei* BFT system. By integrating water quality monitoring with high-throughput sequencing, we aimed to characterize the ecological responses of bacterial communities to peanut shell addition and to clarify how slow-degrading carbon inputs regulate bacterial succession processes, community assembly, and nutrient transformation in BFT systems. This work provides insight into the microecological role of lignocellulosic agricultural residues in aquaculture and offers a basis for improving carbon source management strategies in sustainable shrimp farming.

## 2 Materials and methods

### 2.1 Animal feeding and experimental design

This study was conducted at the Yongxing base of the Zhejiang Fisheries Research Institute. Healthy juvenile shrimp of *Penaeus vannamei* used in the experiment were purchased from the Qingjiang base of Zhejiang Marine Fisheries Research Institute, with an average body length of (0.80 ± 0.10) cm and an average body weight of (1.20 ± 0.12) mg. All the juvenile shrimp were acclimated for 2 days under the same culture conditions.

The experiment lasted for 28 days. The juvenile shrimp were randomly divided into two groups: the control group and the peanut shell–amended group. Each group was set up with three parallel replicates, and each replicate was raised in a 100 L round plastic bucket with an initial stocking density of about 2000 shrimp/bucket. The control group was fed with commercial basic feed (Shenzhen AlphaFeed Company, carbon content 44.20%, nitrogen content 7.04%) throughout the whole process. The peanut shell–amended group received pretreated peanut shells as an exogenous carbon source on the basis of the same amount of basic feed, and its carbon and nitrogen content were according to the literature method (carbon 46.41%, nitrogen 1.14%) (Yao et al., 2015). The target carbon-nitrogen ratio in this study was set at 15:1. The pretreatment of peanut shells included washing with deionized water, drying at 60 °C for 24 h, and grinding through an 80-mesh sieve. According to the carbon-nitrogen ratio regulation model proposed by Avnimelech (1999):


C/N=(M_f×C_f+M_ps×C_ps)/(M_f×N_f+M_ps×N_ps)

where M_f and M_ps represent the dry base mass (g) of the feed and peanut shell, respectively, C_f and C_ps are the carbon content (%), and N_f and N_ps are the nitrogen content (%). After calculation, the amount of peanut shell added to reach the target carbon-nitrogen ratio was about 230% of the feed feeding quality (i.e., the mass ratio M_f: M_ps ≈ 1: 2.3). Finally, the peanut shell powder and the basic feed were mixed and granulated according to this ratio, and the mixture was prepared and used immediately.

During the experiment, the shrimp were fed four times a day at 08:00, 12:00, 15:00, and 20:00. The total daily feed amounted to 5–8% of the shrimp’s body weight, and the feed amount was adjusted every 3 days according to the feeding situation and water quality changes. During the culture period, the water quality conditions were kept stable: the water temperature was controlled at (25 ± 0.5) °C, the salinity was 18‰, the pH was (8.1 ± 0.1), and the dissolved oxygen concentration was always maintained above 5 mg/L through continuous aeration. This study adopted a single-carbon-source experimental design, in which peanut shells were used as the sole exogenous carbon input.

### 2.2 Sample collection

Sampling was performed at 09:00 on days 1, 14, and 28 of the culture. 1,500 mL of water was randomly collected from the middle of each barrel (about 30 cm deep) to ensure the consistency of sample conditions. A disinfected sampler was used to avoid disturbing the bottom sediment. The collected water samples were divided into two parts: one part was used for the detection of chemical indicators of water, and the other part was filtered through a 0.22 μm polycarbonate membrane (Millipore, United States) to collect microorganisms. During the filtration process, pre-filtration membranes were used to remove large particulate impurities. The filter membrane samples were quickly frozen in liquid nitrogen and stored at −80 °C for DNA extraction and subsequent sequencing analysis.

### 2.3 Chemical analysis of water quality

The collected water samples were used to determine ammonia nitrogen (NH₄^+^-N), nitrite nitrogen (NO₂^−^-N), phosphate phosphorus (PO₄^3−^-P), total phosphorus (TP), and chlorophyll a (Chl a). Ammonia nitrogen was determined by Nessler’s reagent spectrophotometry (HJ 535-2009), nitrite nitrogen was determined by spectrophotometry (GB 7493-87), phosphate and total phosphorus were determined by ammonium molybdate spectrophotometry (GB 11893-89), and chlorophyll a was determined by hot ethanol extraction (Jespersen and Christoffersen, 1987).

At the same time, water temperature, pH, dissolved oxygen (DO), and salinity were monitored in real time by a multi-parameter water quality analyzer (YSI ProDSS, model 154,713) to ensure the stability of the aquaculture environment.

### 2.4 DNA extraction and 16S rRNA gene sequencing

Microbial DNA from the water samples was extracted using the HiPure Stool DNA Mini Kit (Magen, China). PCR amplification was performed targeting the V3–V4 region of the 16S rRNA gene using the universal primers 341F (CCTACGGGNGGCWGCAG) and 805R (GACTACHVGGGTATCTAATCC). To reduce amplification bias, each sample was subjected to three independent PCR reactions. The total volume of PCR was 20 μL, and the reaction conditions were pre-denaturation at 95 °C for 3 min, denaturation at 95 °C for 30 s, annealing at 55 °C for 30 s, and extension at 72 °C for 45 s, for a total of 30 cycles, followed by a final extension at 72°C for 10 min. The negative control was set as a blank template to test for contamination. The three PCR products of the same sample were combined and purified using the TaKaRa PCR fragment purification kit. After purification, double-ended sequencing was performed on the Illumina MiSeq platform (2 × 300 bp).

### 2.5 Biostatistics

The raw sequencing data were quality controlled using Dix-seq 1.0 software [refer to Caporaso et al. (2010)], and the UNOISE algorithm was used for denoising and chimera sequence removal, and finally high-quality zero-radius operational taxonomic units (ZOTUs) were obtained. ZOTU sequences were annotated for species classification using the SILVA 138.2 database, and chloroplasts, mitochondria, unclassified archaea and bacteria, and sequences that appeared only once (singletons) were removed. To normalize the sequencing depth, the ZOTUs table was diluted to 26,272 sequences per sample for subsequent analysis.

The *α* diversity indices and *β* diversity indices of the bacterial community were calculated using QIIME software and the “vegan” package in the R language. The Wilcoxon rank sum test was used to analyze the differences between groups in the α diversity index. Principal coordinate analysis (PCoA) was performed based on the Bray-Curtis distance matrix, and the statistical significance of the community structure between groups was tested using permutation multivariate variance analysis (PERMANOVA, number of permutations = 999). The community composition analysis was performed by calculating the relative abundance of phylum, family, and other classification levels, and the groups with an average relative abundance of more than 0.1% were selected as the dominant flora. At the same time, the ZOTUs with an average relative abundance of more than 0.1% were standardized by Z-score to analyze their distribution patterns.

To reveal the interaction between microbial communities and environmental factors, we performed redundancy analysis (RDA) combined with 499 Monte Carlo permutation tests to evaluate the degree of explanation of key water quality parameters (including ammonia nitrogen, nitrite, nitrate, phosphate, and total phosphorus) on community variation. In addition, the Spearman rank correlation coefficient between the dominant flora (family/genus level) and these water quality parameters was calculated to identify significant association patterns.

In terms of functional prediction, based on 16S rRNA gene sequencing data, the PICRUSt2 tool was used to predict the abundance of KEGG orthologous groups (KO) and metabolic pathways. The R package limma was used to screen the KO functions with significant differences between the treatment group and the control group, and the threshold was set to |log₂FC| > 1 and *p* < 0.05. At the same time, the association network of KO and the third-level metabolic pathway (L3) was constructed for visualization analysis.

To quantify the relative contributions of stochastic and deterministic processes in bacterial community assembly, we applied the neutral community model (NCM) described by Sloan et al. (2006). The model evaluates whether the occurrence frequency of zero-radius operational taxonomic units (ZOTUs) in local communities can be predicted from their mean relative abundance in the metacommunity, providing an assessment of the extent to which community assembly follows neutral expectations.

All samples were treated as a single metacommunity. For each ZOTU, its occurrence frequency across samples and its mean relative abundance in the metacommunity were calculated and used as model inputs. The migration rate (*m*) was estimated using nonlinear least-squares fitting and represents the strength of dispersal from the metacommunity to local communities, with higher m values indicating weaker dispersal limitation (Burns et al., 2016). Model performance was evaluated using the coefficient of determination (*R*^2^), which ranges from ≤0 (not fit) to 1 (perfectly fit).

Based on the 95% confidence interval of the model predictions, ZOTUs were classified as neutral (within the confidence interval), above prediction (exceeding the upper limit), or below prediction (below the lower limit). The taxonomic composition of ZOTUs in each category was subsequently examined at the phylum and family levels to identify lineage-specific deviations from neutral expectations.

### 2.6 Statistical analysis

The relationship between bacterial communities and water physicochemical indicators was calculated using Spearman’s rank correlation analysis. The correlation coefficient (*ρ*) and significance level (*p*-value) were calculated using the Hmisc package in the R language environment (version 4.3.1). All *p*-values were corrected for the false discovery rate (FDR) using the Benjamini-Hochberg method. The results with |ρ| > 0.5 and corrected *p* < 0.05 were considered significantly correlated.

All statistical and visualization operations were completed in the R language environment. The physicochemical parameters of water were expressed as “mean ± standard deviation.” The differences between groups were analyzed by an independent sample *t*-test, and the significance level was set as *p* < 0.05.

## 3 Results

### 3.1 The addition of peanut shells significantly reduced the concentration of ammonia nitrogen and nitrite in the water

In this study, the effects of peanut shell amendment on the rearing water of *Penaeus vannamei* were systematically monitored during a 28-day culture experiment. The results of water quality parameter determination showed that the peanut shell–amended treatment significantly changed the dynamics of nitrogen nutrients in the water (Supplementary Figure S1).

Differences in ammonia nitrogen (NH₄^+^-N) concentrations between groups gradually widened over time. On the first day of the experiment, there was no significant difference in the concentration of ammonia nitrogen between the control group and the treatment group (*p* > 0.05). On day 14, the average ammonia nitrogen concentration in the treatment group was lower than that in the control group, but the difference was not significant (*p* > 0.05). At the end of the experiment (day 28), the ammonia nitrogen concentration in the treatment group (2.02 ± 3.44 mg/L) was significantly higher than that in the control group (1.34 ± 0.40 mg/L, *p* < 0.01).

The concentration of nitrite nitrogen (NO₂^−^-N) was also significantly reduced in the treatment group. On day 1, the concentrations in both groups showed no significant difference. On day 14, the nitrite nitrogen concentration in the treatment group (0.35 ± 0.35 mg/L) was significantly lower than that in the control group (2.49 ± 2.28 mg/L) (*p* < 0.05). On day 28, the concentration in the treatment group (28.19 ± 8.62 mg/L) was significantly lower than that in the control group (37.78 ± 5.06 mg/L) (*p* < 0.001). Such a marked reduction in toxic nitrogenous compounds reflects efficient heterotrophic assimilation, a typical feature of biofloc systems supplemented with organic carbon sources (Ayazo Genes et al., 2025).

In contrast, chlorophyll a (Chl a) concentrations showed no significant differences between groups throughout the experiment (*p* > 0.05). The monitoring data on days 1, 14, and 28 all showed that the chlorophyll a concentration in the treatment group and the control group were at the same statistical level.

### 3.2 Analysis of the overall structure of the bacterial community in water

To comprehensively assess the influence of peanut shell addition on the bacterial community, both *α*- and *β*-diversity indices were analyzed.

**Figure 1:**
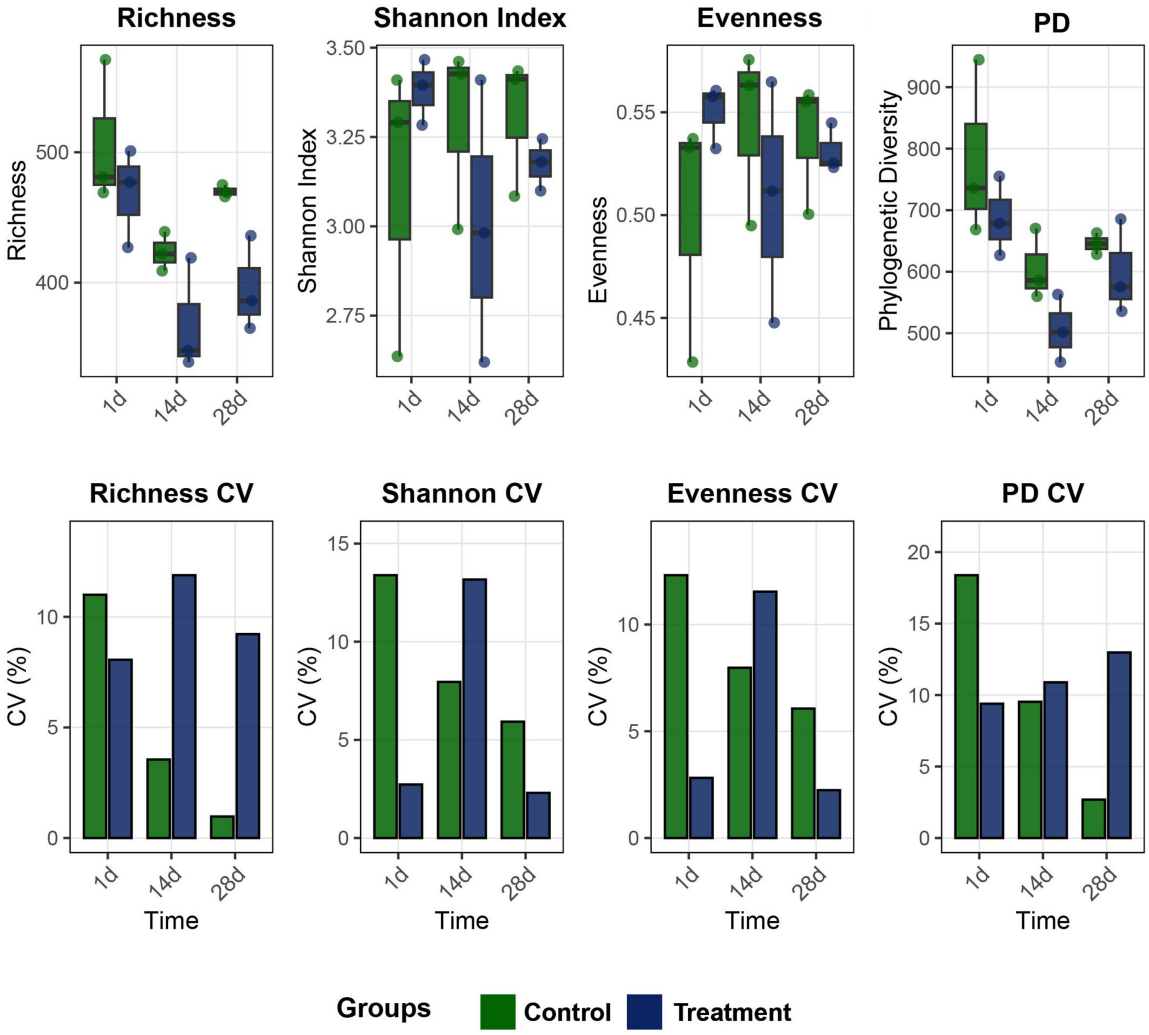
Comparison of *α*-diversity and coefficient of variation (CV%) of the bacterial community in shrimp aquaculture water between the control and peanut-shell-added groups. CV% represents the relative temporal variability of each α-diversity index during the 28 day culture period.

This study analyzed the temporal dynamic effects of treatment on the α diversity of microbial communities (Figure 1). The results showed that the Shannon index and phylogenetic diversity decreased in the early stage of treatment (1 day), indicating that the treatment constituted a short-term ecological disturbance to the community. Subsequently, the diversity indices gradually recovered to the level of the control group at 14 days and 28 days, indicating that they showed recovery of diversity indices over time. Analysis of the coefficient of variation showed that intra-group variability increased at the early stage but later stabilized, suggesting that the community reached equilibrium after the initial disturbance.

In terms of the overall community structure (β diversity), the principal coordinates analysis (PCoA) showed that the sample points of the control group and the peanut shell treatment group were widely overlapped in the sorting space based on the Bray-Curtis distance (Supplementary Figure S2). The sample distribution did not show an obvious clustering trend according to the treatment group, indicating that the bacterial community structure between the two groups was similar as a whole.

The results of the permutational multivariate analysis of variance (PERMANOVA) supported the above observations (Supplementary Table S1). On day 1, the treatment factor explained 21.5% of the community variation (*R*^2^ = 0.215), but the statistical test was not significant (*p*-adj = 0.643). On day 14, the amount of variation explained by the treatment factor decreased to 10.4% (*R*^2^ = 0.104, *p*-adj = 0.900). On day 28, the explanatory power of the treatment factor was 16.1% (*R*^2^ = 0.161, *p*-adj = 0.857). PERMANOVA tests at all three time points showed no significant differences in community structure between groups.

Analysis of similarities (ANOSIM) yielded consistent conclusions, with R values of 0.148 (*p*-adj = 0.500) on day 1, −0.222 (*p*-adj = 0.857) on day 14, and −0.259 (*p*-adj = 0.900) on day 28, further confirming that the differences between groups were smaller than the differences within groups.

Taken together, although the addition of peanut shells caused a short-term increase in the number of species within the treatment group, it did not continuously change the overall diversity level of the community (no difference between groups), nor did it significantly reshape the overall structure of the community (no difference in *β* diversity).

### 3.3 Enrichment of specific organic matter-degrading bacterial groups under peanut shell addition

**Figure 2:**
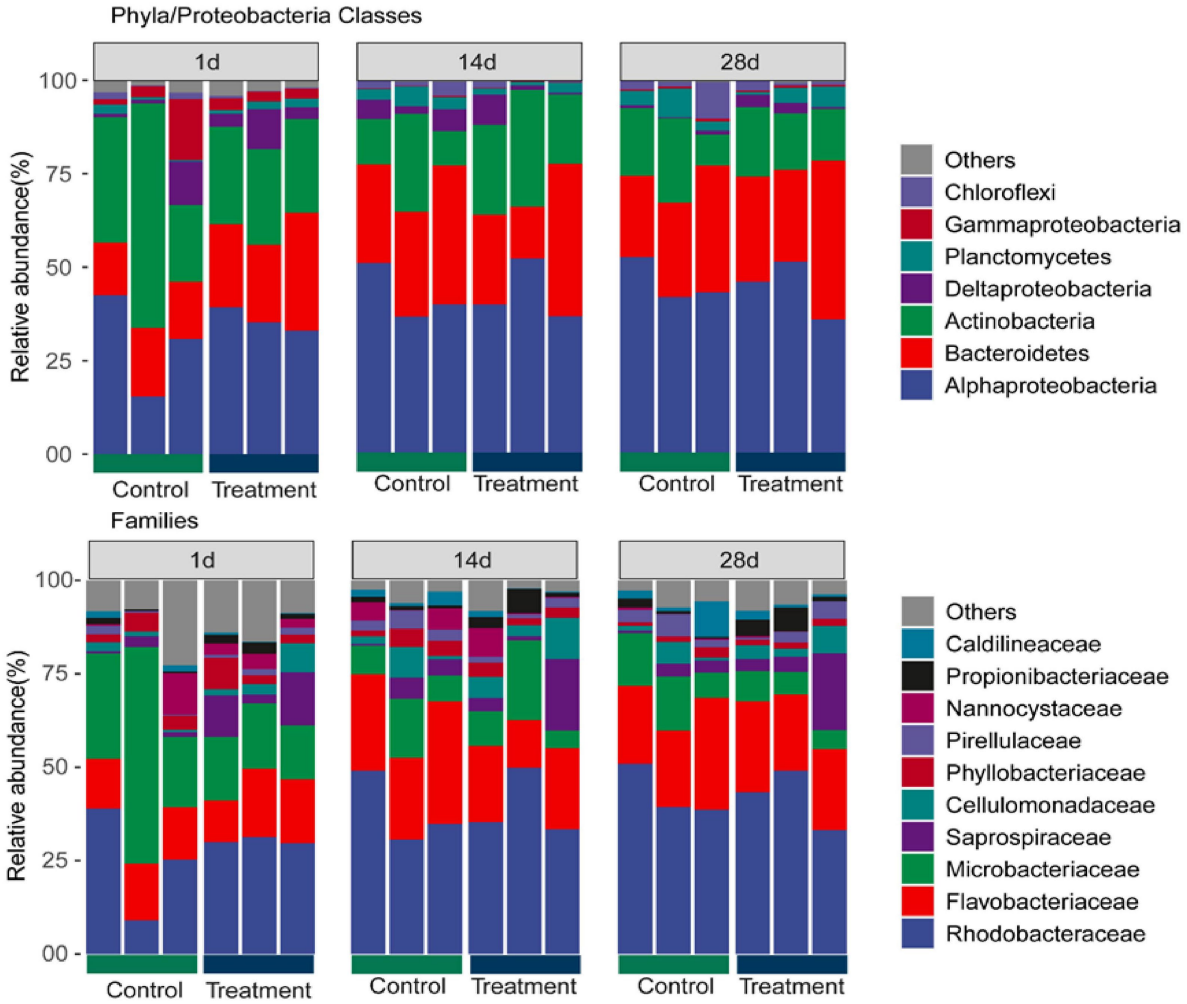
Relative abundances of dominant bacterial phyla/*Pseudomonadota* classes (top) and families (bottom) in rearing water. Only taxa with an average relative abundance > 0.1% across all samples are shown. Data are presented for control and peanut-shell-added groups on days 1, 14, and 28. Differences between groups at each time point were evaluated using the Wilcoxon rank-sum test.

Although the overall structure of the bacterial community (β-diversity) remained stable, relative abundance analysis at the phylum and family levels showed that specific bacterial groups responded to peanut shell–amended treatment (Figure 2; Supplementary Tables S2, S3).

At the phylum level, *Bacteroidetes* showed a trend of enrichment in the treatment group. On day 1, the relative abundance of *Bacteroidetes* in the treatment group (0.248 ± 0.058) was higher than that in the control group (0.160 ± 0.022) (*p* = 0.069). On day 28, the abundance of the treatment group (0.317 ± 0.095) was still higher than that of the control group (0.269 ± 0.062) (*p* = 0.509). In contrast, the abundance of Actinobacteria showed a decreasing trend on day 1, with the abundance of the treatment group (0.256 ± 0.005) lower than that of the control group (0.381 ± 0.202) (*p* = 0.344).

At the family level, the response of the *Leptospiraceae* family was the most obvious. On day 1, the relative abundance of this family in the treatment group (0.092 ± 0.061) was higher than that in the control group (0.015 ± 0.012) (*p* = 0.101). On day 28, the abundance of the treatment group (0.093 ± 0.098) was still higher than that of the control group (0.025 ± 0.015) (*p* = 0.301). In addition, *Propionibacteriaceae* (Day 1: treatment group 0.022 ± 0.008, control group 0.009 ± 0.007) and *Cellulomonadaceae* (Day 1: treatment group 0.041 ± 0.032, control group 0.015 ± 0.010) also showed a trend of being higher than the control group on Day 1. Conversely, *Flavobacteriaceae* were less abundant in the treatment group (0.183 ± 0.048) than in the control (0.268 ± 0.056) on day 14.

### 3.4 The dominant role of environmental factors in the construction of bacterial communities in water

**Figure 3:**
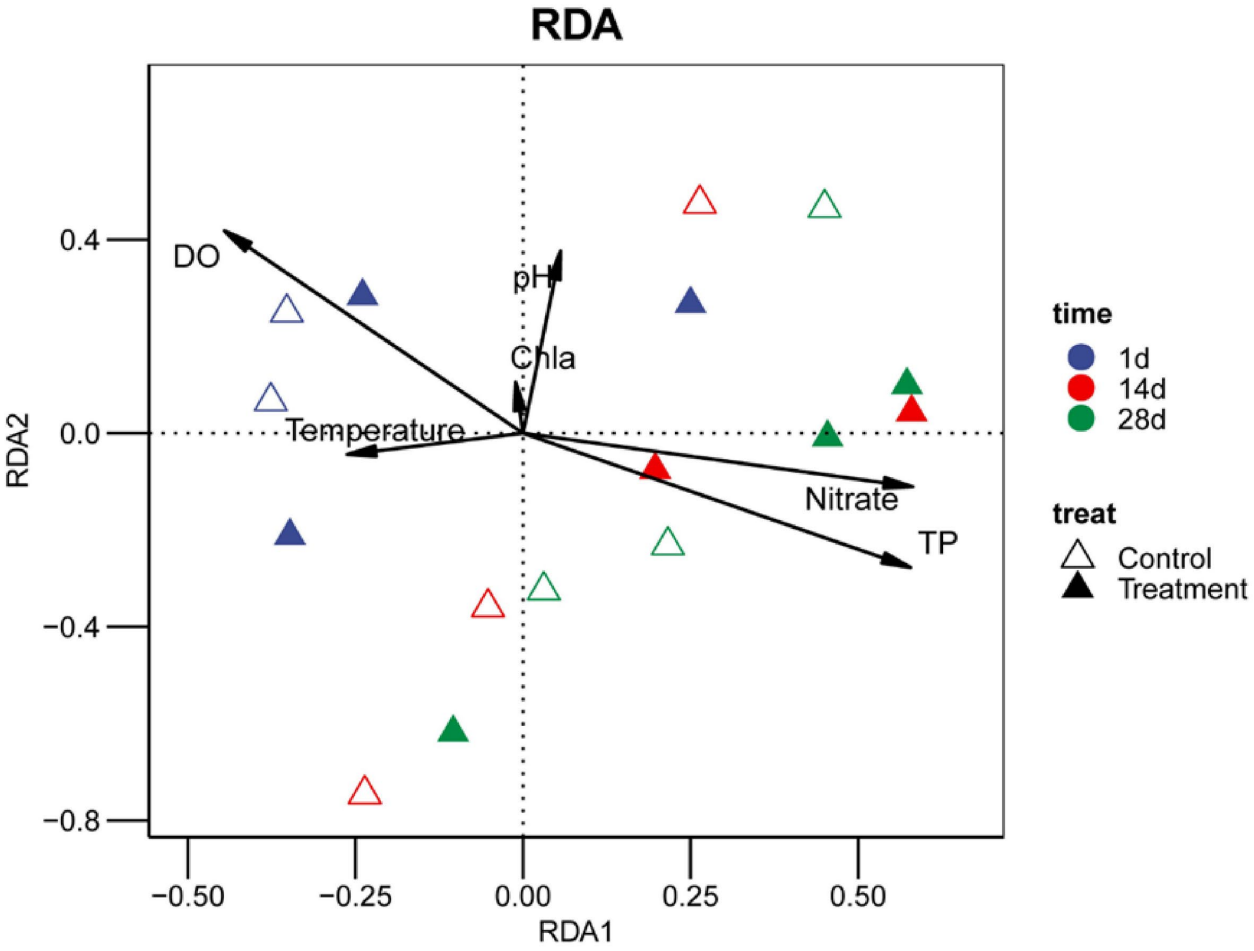
Redundancy analysis (RDA) illustrating relationships between bacterial community composition and environmental variables. Key water-quality parameters (NH_4_^+^–N, NO_2_^−^–N, NO_3_^−^–N, PO_4_^3−^–P, and TP) are shown as explanatory variables. Samples are color-coded by sampling day. Arrows indicate the strength and direction of correlation between environmental factors and ordination axes. The explanatory power (*R*^2^) and statistical significance (*P*) of each parameter are summarized in the accompanying table.

To explore the key environmental factors affecting the construction of bacterial communities in water, we conducted redundancy analysis (RDA). The results showed that all sample points presented a consistent succession direction along the time gradient, from the starting area on the 1st day, through the transition on the 14th day, and finally gathered in the area on the 28th day (Figure 3).

Despite the common temporal succession trend, the peanut shell–amended group samples were always separated from the control group samples in the RDA sorting space during the whole experimental period. On the first day, the treatment group samples were mainly distributed in the negative direction of the RDA1 axis, while the control group samples were relatively scattered. By the 28th day, the two groups of samples formed a more obvious separation pattern along the RDA2 axis, indicating that the treatment effect was enhanced with the passage of time.

The environmental factor fitting analysis showed that among all the water quality parameters measured, phosphate (PO₄^3−^-P) had the highest explanatory power for community variation (*R*^2^ = 0.796, *p* = 0.001), and its vector direction was strongly positively correlated with the RDA1 axis. The explanation of total phosphorus (TP) was the second highest (*R*^2^ = 0.680, *p* = 0.001), and the explanation of nitrate (NO₃^−^-N) was 0.584 (*p* = 0.001). In contrast, the explanatory power of ammonia nitrogen (NH₄^+^-N) was lower (*R*^2^ = 0.348, *p* = 0.030), and the explanatory power of nitrite (NO₂^−^-N) did not reach a significant level.

The distribution of the treatment group samples in the RDA ordination space was closer to the vector direction of phosphate, total phosphorus, and nitrate, especially in the samples on the 28th day; this association pattern was more obvious. The first two RDA axes together explained 58.6% of the total variation in community structure, with RDA1 and RDA2 accounting for 41.2 and 17.4%, respectively.

### 3.5 Integrated temporal succession of dominant zOTUs and their correlations with environmental factors

**Figure 4:**
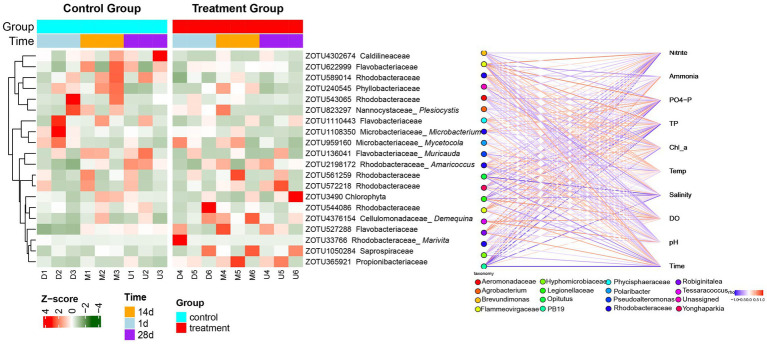
Abundance patterns of dominant zOTUs and their correlations with environmental parameters. Left: Clustered heatmap showing Z-score–standardized relative abundances of dominant zOTUs across samples. Right: Structured correlation network illustrating significant Spearman correlations (|*ρ*| ≥ 0.6, adjusted *p* < 0.05; Benjamini–Hochberg correction). Red and blue lines denote positive (ρ ≥ 0.6) and negative (ρ ≤ −0.6) correlations, respectively, with line intensity proportional to correlation strength.

To better understand how the microbial community responded to peanut shell amendment, we examined dominant zOTUs (average relative abundance > 0.1%) through a Z-score–standardized heatmap combined with Spearman correlation analysis (Figure 4). The heatmap (left panel) clearly illustrated that the abundance profiles of dominant zOTUs changed dynamically over time. At the start of the experiment (day 1), the overall composition of zOTUs was comparable between the control and treatment groups, and the clustering pattern mainly reflected individual variation rather than treatment effects. As the rearing period progressed, the two groups gradually diverged. By days 14 and 28, samples from the peanut-shell-amended group began to cluster separately according to sampling time, suggesting that temporal progression and the addition of peanut shells acted together in shaping the subtle composition of the bacterial community.

Several clear succession trends were observed. zOTUs affiliated with *Spirochaetaceae* maintained relatively high abundance in the treatment group on days 14 and 28, while members of *Flavobacteriaceae* showed marked enrichment during the middle and late stages. In contrast, multiple zOTUs assigned to *Rhodobacteraceae* displayed a consistent decline in relative abundance over time in the treatment group. Calculation of within-group Bray–Curtis dissimilarity further supported this pattern: community differences within the treatment group on day 28 (0.32 ± 0.08) were smaller than those in the control (0.45 ± 0.11), suggesting reduced within-group variation Together, these zOTU-level patterns indicate a time-dependent, directional succession of specific taxa while preserving overall structural stability.

The correlation network (right panel) provided additional insight into how these taxa were associated with environmental conditions. Significant Spearman correlations (|*ρ*| ≥ 0.6, adjusted *p* < 0.05) indicated that both nitrogen and phosphorus variables were key drivers of bacterial community assembly. Among the phosphorus-related taxa, several OTUs belonging to *Phycisphaeraceae* showed strong positive correlations with phosphate (PO₄^3−^–P) and total phosphorus (TP) (*ρ* = 0.62–0.78). Similarly, the genera *Robiginitalea* and *Polaribacter* were moderately positively correlated with phosphate concentrations (ρ = 0.45–0.58), suggesting that phosphorus enrichment may have favored these heterotrophic lineages.

For nitrogen-associated taxa, several OTUs within *Rhodobacteraceae* were negatively correlated with ammonium (NH₄^+^–N) (ρ = −0.51 to −0.69), and *Brevundimonas* showed a negative correlation with nitrite (NO₂^−^–N) (ρ = −0.43). In contrast, multiple OTUs affiliated with *Flavobacterium* were positively correlated with nitrate (NO₃^−^–N) (ρ = 0.48–0.61), implying potential roles in nitrification or nitrate utilization.

Responses to dissolved oxygen (DO) varied among taxa: *Hyphomicrobiaceae* was positively correlated with DO (ρ = 0.52), whereas *Aeromonadaceae*, *Legionellaceae*, and *Tessaracoccus* were negatively correlated (ρ = −0.46 to −0.57). In addition, several taxa closely linked to peanut shell addition—*Agrobacterium*, *Opitutus*, and *Pseudoalteromonas*—were identified as core members that correlated significantly with multiple environmental factors simultaneously. These relationships revealed two relatively independent yet interconnected modules within the network: one dominated by taxa positively associated with phosphate, and the other by taxa negatively associated with nitrogenous compounds.

In summary, Figure 4 illustrates that peanut shell addition led to directional changes in dominant zOTUs over time, particularly the enrichment of *Flavobacteriaceae* and *Spirochaetaceae* and the decline of *Rhodobacteraceae*. Correlation analysis further revealed distinct associations between specific taxa and key environmental factors, including nitrogen, phosphorus, and dissolved oxygen.

### 3.6 Peanut shell-induced metabolic function remodeling of microorganisms from basic synthesis to environmental collaboration

**Figure 5:**
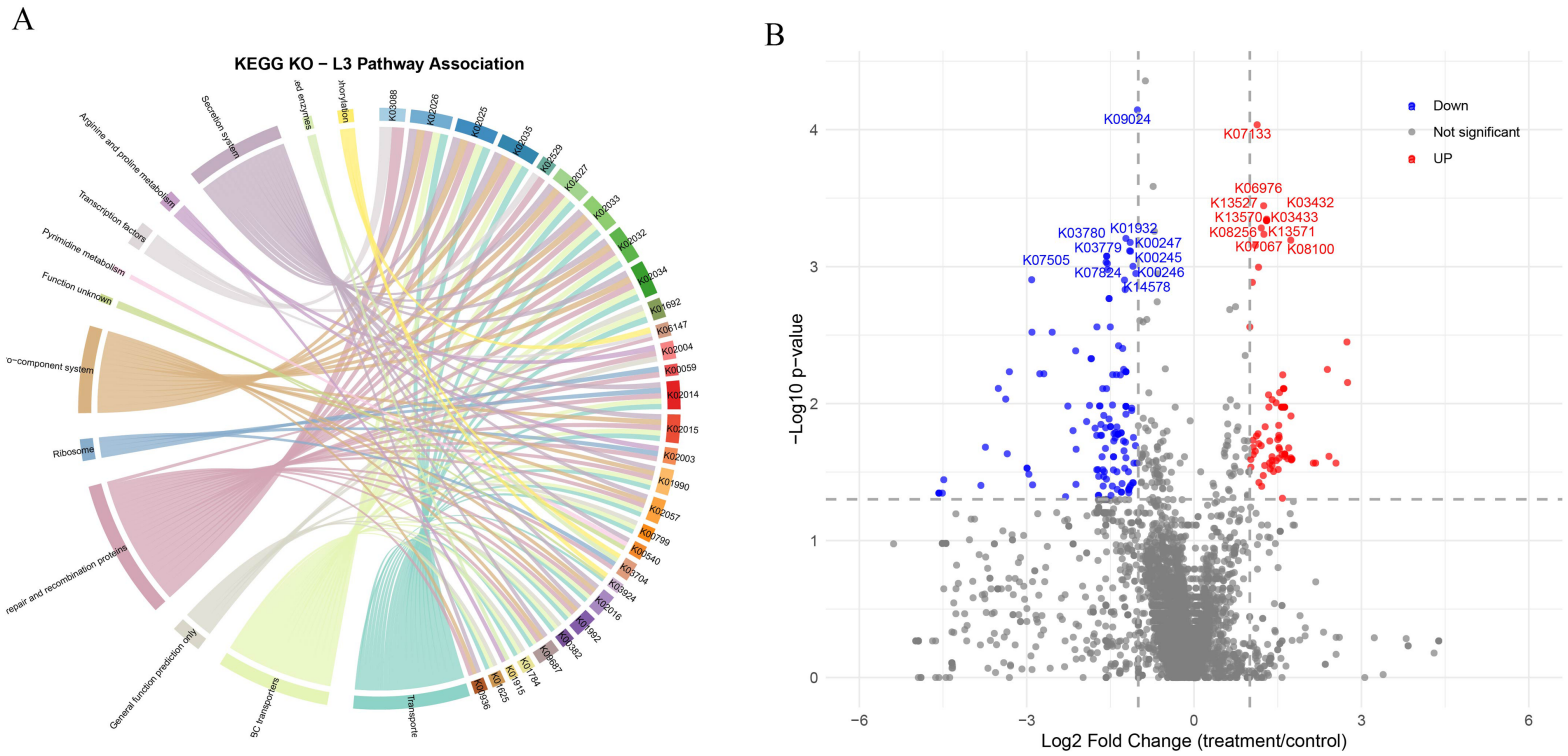
Differential functional profiles of the bacterial community in response to peanut-shell addition. **(A)** Chord diagram showing associations between significantly altered KEGG orthology (KO) groups and their dominant level-3 metabolic pathways. Ribbon width represents the cumulative abundance of KOs associated with each pathway. **(B)** Volcano plot of differentially abundant KOs between peanut-shell-added and control groups. Each point represents a KO; red and blue indicate significantly up- and down-regulated KOs, respectively (|log_2_FC| > 1, adjusted *p* < 0.05). Key functional categories are annotated. The functional annotations of the KO terms shown in the figure are provided in Supplementary File 2.

Functional prediction analysis based on PICRUSt2 showed that peanut shell treatment caused extensive reshaping of the KEGG Orthology (KO) functional spectrum of the water microbial community (Figure 5). Chord diagram (Figure 5A) analysis showed that the pathways of “carbohydrate metabolism,” “amino acid metabolism,” and “energy metabolism” established the most intensive connection with the core KO, constituting the core hub in the network.

Volcano plot analysis (Figure 5B) (threshold set at |log₂FC| > 1 and *p* < 0.05) identified a large number of differential KOs. In the treatment group, the significantly up-regulated KOs mainly included genes related to quorum sensing (K09024, K07133), the two-component system (K06976), the bacterial secretion system (K03432, K03433, K13571), peptidase (K08256), and flagellar assembly (K13527, K13570).

Meanwhile, the significantly down-regulated KOs were mainly enriched in the related pathways such as amino acid biosynthesis (K03780, K01932, K03779, K00247, K07505, K00245, K07824, K00246), vitamin metabolism (K14578), and oxidative phosphorylation (K07824). The number of down-regulated KOs exceeded that of up-regulated KOs, with 131 down-regulated and 87 up-regulated KOs among the 218 significantly different KOs.

Functional category statistics showed that the functional categories related to bacterial motility, chemotaxis, quorum sensing, and membrane transport system dominated the significantly down-regulated KOs. The functional categories related to complex organic matter degradation, nitrification, and denitrification were significantly enriched in the significantly up-regulated KOs.

### 3.7 Effects of peanut shell addition on bacterial community assembly

To assess bacterial community assembly processes, the neutral community model (NCM) was applied to both the control and peanut-shell-treated groups. Overall, the model showed a good fit to the observed data in both groups, indicating that community assembly patterns were largely consistent with neutral expectations.

**Figure 6:**
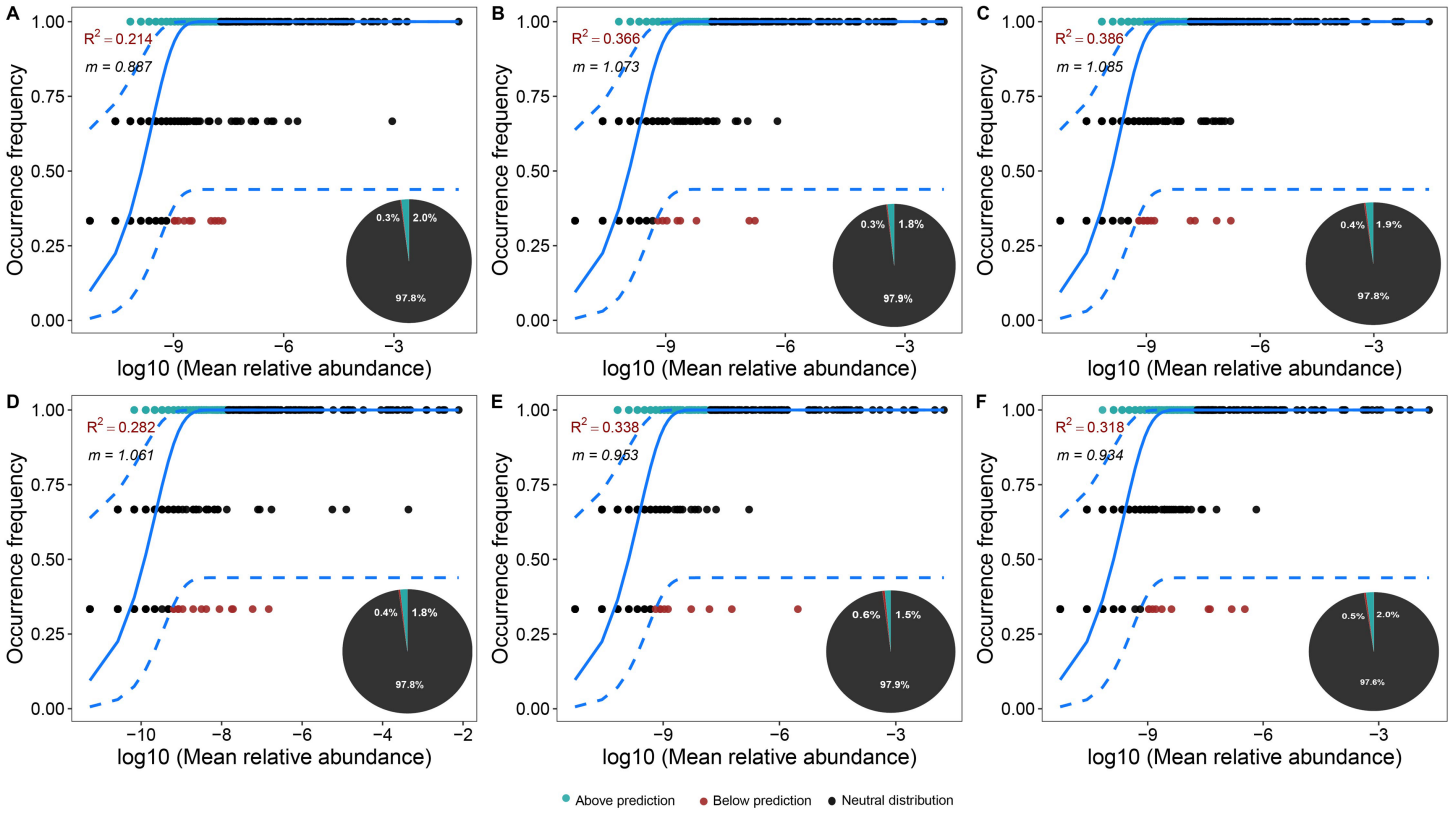
Neutral model (NCM) fit for control **(A–C)** and treated **(D–F)** groups at 1, 14, and 28 days. Points show OTU occurrence vs. mean relative abundance; curves depict model fit and 95% CI. OTU categories: neutral (circles), above (triangles), below (squares). *R*^2^ and *m* values are given per plot, with category proportions (%) noted.

Neutral community model (NCM) analysis showed that bacterial communities in both the control and peanut-shell-treated groups were well fitted by the neutral model, indicating that their assembly patterns were largely consistent with neutral expectations. Distinct temporal patterns in the migration rate (*m*) were observed between the control and treatment groups (Figure 6). Over the experimental period, m values in the control group showed a gradual increase, whereas m values in the treatment group exhibited a decreasing trend. Except for the initial stage (1 d), m values in the control group were consistently higher than those in the treatment group at 14 d and 28 d During the middle and late stages, persistent differences in migration rates (*m*) were observed between the control and peanut-shell-treated groups.

**Figure 7:**
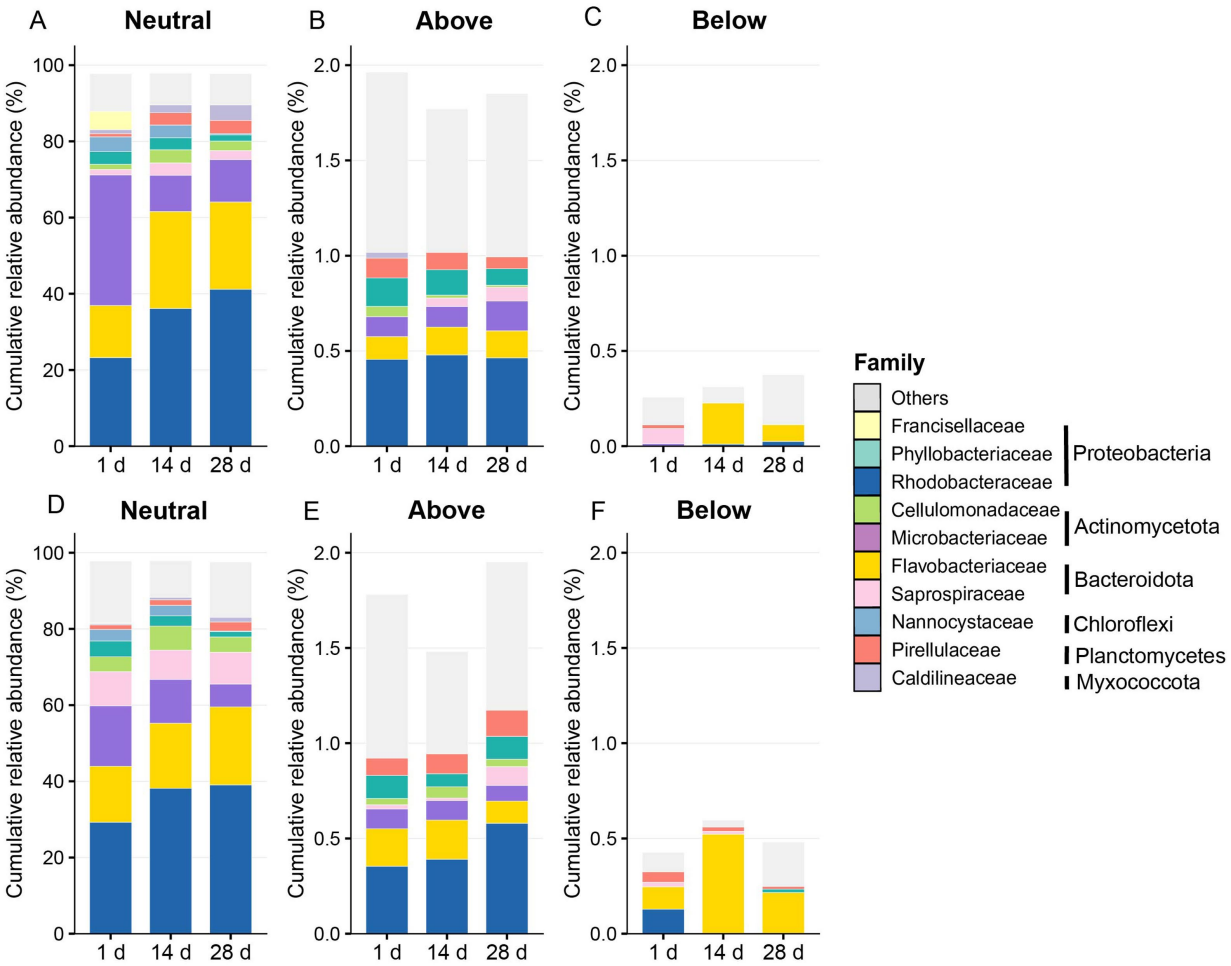
Distribution of taxa under the neutral model in control and treatment groups. **(A–C)** Control group: relative abundances of OTUs classified as neutral **(A)**, above prediction **(B)**, and below prediction **(C)**. **(D–F)** Peanut-shell-treated group: relative abundances of OTUs classified as Neutral **(D)**, above prediction **(E)**, and below prediction **(F)**. Only the top 10 most abundant bacterial families are shown, with all remaining taxa grouped as “Others.” Colored bars represent family-level taxonomic composition, while vertical annotations in the legend indicate the phylum to which each family belongs.

In both groups, the proportion of neutrally distributed OTUs increased over time (Figure 7). Meanwhile, the proportions of above- and below-prediction OTUs varied across sampling time points, indicating differences in the degree to which specific taxa deviated from neutral model predictions under different temporal and treatment conditions. Among non-neutral OTUs, below-prediction taxa in the control group were mainly affiliated with *Flavobacteriaceae*, and their relative composition remained comparatively stable across time points. In contrast, *Flavobacteriaceae* accounted for a higher proportion of below-prediction OTUs in the treatment group. Differences were also observed in the composition of above-prediction OTUs between the two groups, with *Rhodobacteraceae* contributing more substantially to the above-prediction fraction in the treatment group, while their relative contribution was lower in the control group. In addition, within neutrally distributed OTUs, the control group exhibited an increasing proportion of bacteria associated with microalgae over time.

Overall, comparative analysis between the control and treatment groups showed clear differences in the composition of above-prediction OTUs, whereas changes in below-prediction OTUs were more pronounced in the treatment group. Notably, within the below-prediction fraction, the relative degree of deviation associated with *Flavobacteriaceae* was lower in the control group than in the treatment group. Taken together, neutral community model results indicate that community parameters and patterns of deviation from neutral predictions differed across time points and treatment conditions.

## 4 Discussion

### 4.1 Peanut shell amendment does not alter overall community assembly but reshapes lineage-specific distribution patterns

Comparative community analyses showed that peanut shell amendment did not significantly alter the overall microbial community structure or assembly processes with dominant stochastic processes relative to the control throughout the culture period. Similarly, *β*-diversity analysis revealed no significant separation between the treatment and control groups, indicating that peanut shell addition did not induce obvious community turnover or reconstruction of the overall community structure (Supplementary Figure S2; Supplementary Table S1). Consistently, those insignificant changes between the treatment and control groups at the overall community level suggest that peanut shells imposed only weak environmental filtering and did not fundamentally shift the underlying community assembly framework.

This low level of disturbance contrasts with the strong restructuring effects reported for other carbon sources in biofloc systems. Previous studies have shown that readily degradable carbon inputs such as glucose, sucrose, and molasses can rapidly stimulate copiotrophic bacterial growth, strengthen deterministic selection, and induce pronounced shifts in community composition and assembly processes (Crab et al., 2012; Guo et al., 2022; Wei et al., 2016). Similarly, some lignocellulosic agricultural residues have been reported to directionally enrich specific cellulose-degrading taxa (e.g., *Cellulomonas* and *Clostridium*) (Chen et al., 2024; Lynd et al., 2002; Wu et al., 2022), thereby substantially modifying community composition through resource-driven selection. In contrast, peanut shell amendment in the present study did not induce pronounced species replacement, and the overall community composition remained largely comparable to that of the control in terms of composition, structure, and assembly processes, indicating that it functioned as a low-disturbance regulatory carbon source rather than a strong driver of community restructuring.

Although overall community patterns remained consistent, the peanut shell amendment significantly influenced distribution patterns of rare taxa (0.3–2%) in neutral community model. Those taxa without prediction of NCM categories shifts in the relative proportions was indicated selective responses of specific bacterial groups to the peanut-shell-amended environment. Taxa classified as “Above prediction” increased in relative abundance in the treatment group, particularly members of *Rhodobacteraceae*, suggesting positive selection under the slow-release carbon regime (Dang and Lovell, 2016). In contrast, taxa classified as “Below prediction,” mainly affiliated with *Flavobacteriaceae*, showed reduced representation under peanut shell treatment, indicating environmental filtering or competitive exclusion within the modified ecological niche (Fierer et al., 2007). These results demonstrate that peanut shells reshaped community composition through lineage-level selection without disrupting the overall stochastic assembly framework.

Lineage-level changes were further accompanied by shifts in microbial ecological strategies. Temporal dynamics of community composition suggested a gradual transition in dominant resource utilization strategies, characterized by the early proliferation of fast-growing opportunistic taxa followed by enrichment of microorganisms capable of efficiently utilizing complex organic substrates. At the phylum level, *Bacteroidetes* showed continuous enrichment in the treatment group, which is consistent with their ability to degrade complex polysaccharides such as cellulose and hemicellulose and their typical K-strategy characteristics (Fierer et al., 2007). At the family level, *Saprospiraceae* and *Rhodobacteraceae* became increasingly dominant during the middle and late culture stages, reflecting competitive advantages under sustained resource supply (Dang and Lovell, 2016). The temporal pattern of *α*-diversity, characterized by an initial decline followed by recovery and community reorganization, further supports this succession process, suggesting early community adjustment followed by competitive sorting of adapted taxa. These observations collectively indicate that peanut shell amendment promoted a gradual shift toward K-strategy characteristics while maintaining overall community structural consistency (Allison and Martiny, 2008). The observed lineage-specific selection and ecological strategy shift are likely associated with the physicochemical properties of peanut shells as a slow-release lignocellulosic carbon source. Their complex structural components require cooperative microbial degradation and provide sustained resource input, which may reduce strong competitive exclusion and allow coexistence and succession of multiple microbial groups (Tilman, 2020). Consequently, peanut shells appear to regulate microbial community composition through gradual resource-driven selection rather than abrupt environmental perturbation.

Collectively, these findings demonstrate that peanut shell amendment does not cause wholesale restructuring of microbial community assembly but instead modulates distribution patterns of rare abundance taxa and ecological strategies under a predominantly stochastic assembly regime. This low-disturbance regulatory effect distinguishes peanut shells from readily degradable carbon sources and explains the limited structural divergence of the microbial community observed in the peanut-shell-amended system.

### 4.2 Phosphate and nitrate were key environmental factors driving the community construction of water bacterial taxa

The addition of peanut shells not only promoted the succession of bacterial communities but also significantly changed the physical and chemical characteristics of the water environment. The results showed that the concentrations of ammonia nitrogen (NH₄^+^-N) and nitrite nitrogen (NO₂^−^-N) in the water body of the treatment group were significantly lower than those of the control group in the middle and late stages of culture (Supplementary Figure S1), indicating that the intervention of peanut shells significantly affected the nitrogen dynamics of the system.

This change was likely associated with enhanced activity of heterotrophic and organic matter–degrading bacteria, which may have accelerated nitrogen removal from the water (Emerenciano et al., 2013). Meanwhile, the concentration of nitrate nitrogen (NO₃^−^-N) in the treatment group also showed a downward trend in the middle and late stages, reflecting the acceleration of the nitrification–denitrification process in the system and the improvement of nitrogen removal efficiency (Kuypers et al., 2018). Thus, peanut shells enhanced nitrogen cycling by continuous organic carbon supply and reshaped nitrogen transformation pathways.

Furthermore, Spearman correlation analysis was used to explore links between water quality and bacteria (Figure 4). And those results showed that *Rhodobacteraceae* taxa were also significantly negatively correlated with the concentration of ammonia and nitrogen, and negative correlation between the genus *Brevundimonas* and nitrite. They were similar to microbial groups associated with microbiologically influenced corrosion (MIC) in marine environments (Dang et al., 2011) and microbial nitrogen-cycling networks in natural environments (Kuypers et al., 2018), suggesting that *Rhodobacteraceae* group may play an important role in the process of ammonia nitrogen removal and *Brevundimonas* could be involved in the further transformation of nitrite. In addition, the *Phycisphaeraceae* taxa were significantly positively correlated with phosphate (PO₄^3−^-P) concentration in both this work and results of adding in shrimp culture (An et al., 2025), indicating that the abundance change of this group was closely related to the phosphate dynamics in the water. It is worth noting that the positive correlation between nitrifying bacteria (such as *Nitrospira*) and nitrate concentration reflects the enhancement of nitrification in peanut shells system, thus promoting the further oxidation and transformation of nitrogen (Daims et al., 2015). These results together suggest that the peanut shell–amended system could improve water purification efficiency and influence system functioning through interactions between functional bacteria and nutrient dynamics.

To further investigate the functional basis of these microbial responses, we performed PICRUSt2-based functional prediction. Although the overall community structure remained stable, functional prediction (PICRUSt2) indicated that peanut shells induced a subtle yet profound reprogramming at the functional level. Compared with the control, the microbial community in the treatment group showed a shift in metabolic strategy (Figure 5). Genes related to basic anabolism, such as amino acid biosynthesis and oxidative phosphorylation, were down-regulated, suggesting a shift toward more energy-efficient metabolism under the abundant exogenous carbon provided by peanut shells (Roller et al., 2016). Meanwhile, functions related to environmental adaptation and microbial cooperation were up-regulated. Enhanced expression of quorum-sensing and two-component system genes indicates increased microbial synergy (Papenfort and Bassler, 2016), while up-regulated bacterial secretion systems, peptidases, and flagella assembly support extracellular enzyme secretion, macromolecular organic matter decomposition, and substrate colonization (Fierer et al., 2012). Additionally, genes involved in complex organic matter degradation, nitrification, and denitrification were enriched, suggesting improved nitrogen transformation and carbon cycling in the peanut shell system. It should be noted that the functional profiles reported in this study were inferred using PICRUSt2 based on 16S rRNA gene sequences. Consequently, the functional shifts described here should be interpreted as indicative trends in metabolic potential rather than definitive evidence of functional activity. Future studies integrating shotgun metagenomics or transcriptomic validation would further strengthen the mechanistic interpretation of microbial functional responses to peanut shell amendment (Franzosa et al., 2015).

Redundancy analysis (RDA) further revealed the main environmental factors driving community construction. Among all the measured indicators, phosphate (PO₄^3−^-P) had the strongest explanatory power for the bacterial community structure (*R*^2^ = 0.796, *p* = 0.001), followed by nitrate (NO₃^−^-N) (*R*^2^ = 0.642, *p* = 0.002), and total phosphorus (TP) and dissolved oxygen (DO) also had a certain impact (Figure 3; Table 1). The addition of peanut shells primarily altered phosphate and nitrate dynamics, and the two jointly shaped the environmental background of community succession through the coupling with the nitrogen cycle process (An et al., 2025). Interactions between nitrogen and phosphorus availability are well recognized as pivotal regulators of microbial community structure and function (Wang et al., 2020).

**Table 1:** Results of the redundancy analysis (RDA) showing the explanatory power and statistical significance of the environmental variables.

Variable	*R* ^2^	*p*
Nitrate	0.584	0.001^***^
Ammonium	0.348	0.030^*^
PO_4_^+^-P	0.796	0.001^***^
TP	0.680	0.001^***^
Chl a	0.073	0.509
Temperature	0.045	0.720
Salinity	0.679	0.001^***^
DO	0.493	0.003^**^
pH	0.010	0.942

In summary, the addition of peanut shells changed the physical and chemical conditions of the water body, especially the concentration and transformation process of nitrogen and phosphorus nutrients and constructed two-way feedback between the bacterial community structure and ecological function. Correlation analysis revealed the functional relationship between specific bacterial groups and water quality indicators, and redundancy analysis further determined the key role of phosphate and nitrate in community construction. Combined with the results of the previous part, it could be seen that while peanut shells promote the colonization of K-type functional groups, their regulation mechanism is realized through the linkage of “environmental factors-community structure-ecological function.” The regulation of the availability of phosphate and nitrate by peanut shells shapes the resource response strategy of the bacterial community and lays the foundation for subsequent community adjustment and enhanced functional efficiency. The next section will further explore this process from the perspective of community stability and functional response.

### 4.3 Peanut shells potentially regulate microbial community taxa turnover without overall structural restructuring

As an exogenous carbon source, peanut shells exerted only a minor influence on the overall taxonomic structure of the bacterial community in the rearing water. However, functional prediction analysis indicated that peanut shell amendment was associated with changes in metabolic functional profiles despite limited structural divergence of the community. This pattern of structural consistency accompanied by functional adjustment suggests that peanut shells may act as a mild regulator of microbial ecological function in BFT systems.

The similar community structure between treatment and control groups observed in this study, despite detectable functional shifts, can be explained by the concept of functional redundancy in microbial communities, whereby distinct taxonomic assemblages can perform similar ecosystem functions, maintaining overall functional capacity despite compositional variation (Louca et al., 2018). In contrast, previous studies have shown that readily degradable carbon sources typically induce strong taxonomic restructuring in BFT systems. For example, sucrose addition has been reported to favor fast-growing opportunistic taxa and cause pronounced shifts in microbial community composition (Guo et al., 2022). Comparative analyses of glucose, sucrose, and starch further demonstrated that different carbon sources produced markedly distinct community structures, with glucose significantly reducing species richness and enriching potential opportunistic taxa such as Aeromonas (Wei et al., 2016). Similarly, molasses amendment has been shown to rapidly promote floc formation while substantially altering community composition through directional enrichment of specific bacterial groups (Zhang et al., 2024). These findings indicate that readily available carbon sources often impose strong selective pressure and drive rapid compositional shifts.

In contrast, peanut shell amendment in the present study did not induce pronounced species replacement, and overall community composition remained largely comparable to that of the control (Supplementary Figure S2; Supplementary Table S1). This pattern is likely associated with the physicochemical characteristics of peanut shells as a slow-degrading lignocellulosic carbon source. Their complex structural components, such as cellulose and lignin, require cooperative microbial degradation and enable gradual carbon release (Korenblum et al., 2016), which may create heterogeneous ecological niches that facilitate coexistence and succession while mitigating strong competitive exclusion (Liu et al., 2025). As a result, peanut shells appear to regulate microbial functional potential and taxonomic turnover without triggering major structural reorganization (Louca et al., 2018).

Rather than indicating a shift in the overall assembly framework, peanut shell addition was associated with lineage-specific deviations from neutral expectations. Taxa classified as “Above prediction,” particularly members of *Rhodobacteraceae*, were consistently enriched under peanut shell treatment. These taxa are widely recognized for their roles in organic matter degradation, nutrient cycling, and microbial interactions (Dang and Lovell, 2016; Wagner-Döbler et al., 2010), suggesting potential contributions to community metabolic functions. In contrast, taxa classified as “Below prediction,” mainly affiliated with *Flavobacteriaceae*, showed reduced representation under treatment. Their abundance in the present system was positively associated with unfavorable water quality indicators, suggesting selective constraint under peanut shell amendment.

Temporal variation in migration rate (*m*) further indicated that stochastic processes remained an important component of community assembly throughout the culture period, while peanut shell amendment was associated with lineage-level selective responses of rare abundance taxa rather than overall community restructuring (Figures 6, 7). A relatively lower migration rate under peanut shell amendment may reflect differences in dispersal dynamics and a potential enhancement of local environmental filtering; however, this interpretation should be regarded as a statistical inference derived from model fitting rather than direct mechanistic evidence of deterministic dominance (Sloan et al., 2006). By linking NCM outputs with taxonomic composition and predicted functional profiles, these results suggest that low-disturbance carbon inputs may regulate microbial functional potential through gradual compositional adjustment.

Nevertheless, this study primarily focused on the ecological effects of pretreated peanut shells on microbial communities. Because this study employed a single-carbon-source design, the findings should be interpreted primarily as mechanistic insights into microbial ecological regulation rather than direct comparisons among different carbon sources. Raw peanut shells may contain secondary metabolites, such as polyphenols and tannins, which could potentially leach into the culture water and influence microbial activity or shrimp physiology (Monlau et al., 2014). During lignocellulosic biomass degradation, aromatic phenolic compounds may be released and interfere with microbial metabolism when present at sufficiently high concentrations (Chen et al., 2020; Lee et al., 2015; Zhang et al., 2024). Although the pretreatment procedures (washing, drying, and grinding) in this study may reduce the presence of polyphenols and tannins, the release dynamics and potential long-term effects remain unclear. Future studies should therefore quantify these compounds and evaluate their ecological and physiological impacts (Jönsson and Martín, 2016). In addition, comparative experiments involving multiple lignocellulosic substrates would help determine whether different materials exhibit distinct ecological regulatory effects in BFT systems.

Overall, the peanut shell amendment was associated with functional adjustment of microbial communities through gradual resource-driven processes while causing limited structural divergence. These findings suggest that slow-release lignocellulosic inputs may provide a feasible strategy for modulating microbial ecological function in shrimp culture systems.

## 5 Conclusion

In conclusion, these findings demonstrate that peanut shells can drive a gentler microbial response pattern in *P. vannamei* BFT systems, with insignificant variation in community structure alongside predicted metabolic remodeling associated with improved water quality and nutrient transformation in rearing water during the culture period. While this limited impact on microbial community assembly selectively enriched potential beneficial taxa (e.g., *Rhodobacteraceae*) and functions associated with complex organic matter degradation and nitrogen removal, suggesting potential contributions to water purification processes. This low-disturbance regulation strategy offers a practical approach for utilizing abundant agricultural waste to enhance the sustainability of shrimp culture while reducing the environmental footprint.

## Data Availability

All the amplicon sequencing data were deposited in the CNCB (China National Center for Bioinformation, https://ngdc.cncb.ac.cn/) with the BioProject accession number PRJCA054896 and dataset accession number CRA040387. The data are publicly accessible at https://ngdc.cncb.ac.cn/gsa/browse/CRA040387.

## References

[ref1] Allison S. D. Martiny J. B. H. (2008). Resistance, resilience, and redundancy in microbial communities. Proc. Natl. Acad. Sci. 105, 11512–11519. doi: 10.1073/pnas.0801925105, 18695234 PMC2556421

[ref2] An S. Li J. Du J. Feng L. Zhang L. Zhang X. . (2025). Coupled nitrogen and phosphorus cycles mediated by coordinated variations of functional microbes in industrial recirculating aquaculture system. Water Res. 280:123726. doi: 10.1016/j.watres.2025.123726, 40305950

[ref3] Avnimelech Y. (1999). Carbon/nitrogen ratio as a control element in aquaculture systems. Aquaculture 176, 227–235. doi: 10.1016/S0044-8486(99)00085-X

[ref4] Ayazo Genes J. E. Holanda M. Lara G. (2025). Effects of different organic carbon sources on water quality and growth of *Mugil cephalus* cultured in biofloc technology systems. Fishes 10:427. doi: 10.3390/fishes10090427

[ref9005] Burns A. R. Stephens W. Z. Stagaman K. Wong S. Rawls J. F. Guillemin K. . (2016). Contribution of neutral processes to the assembly of gut microbial communities in the zebrafish over host development. The ISME Journal, 10, 655–664. doi: 10.1038/ismej.2015.142 26296066 PMC4817674

[ref5] Caporaso J. G. Kuczynski J. Stombaugh J. Bittinger K. Bushman F. D. Costello E. K. . (2010). QIIME allows analysis of high-throughput community sequencing data. Nat. Methods 7, 335–336. doi: 10.1038/nmeth.f.303, 20383131 PMC3156573

[ref7] Chen X. Zhai R. Li Y. Yuan X. Liu Z. H. Jin M. (2020). Understanding the structural characteristics of water-soluble phenolic compounds from four pretreatments of corn Stover and their inhibitory effects on enzymatic hydrolysis and fermentation. Biotechnol. Biofuels 13:44. doi: 10.1186/s13068-020-01686-z, 32175010 PMC7065323

[ref9003] Chen H. Luo Y. Y. Zeng G. Q. Zhang P. L. Peng R. B. Jiang X. M. . (2022). Effect of adding microalgae to white leg shrimp culture on water quality, shrimp development and yield. Aquac. Rep. 22, 100916. doi: 10.1016/j.aqrep.2021.100916

[ref6] Chen C. Jiang Y. Ren Z. Li M. Wang F. Shan H. (2024). Effects of bagasse as a carbon source on biofloc formation, water quality, and microbial community structure in shrimp culture system. Environ. Sci. Pollut. Res. Int. 31, 42144–42159. doi: 10.1007/s11356-024-33928-0, 38862800

[ref8] Crab R. Avnimelech Y. Defoirdt T. Bossier P. Verstraete W. (2007). Nitrogen removal techniques in aquaculture for a sustainable production. Aquaculture 270, 1–14. doi: 10.1016/j.aquaculture.2007.05.006

[ref9] Crab R. Defoirdt T. Bossier P. Verstraete W. (2012). Biofloc technology in aquaculture: beneficial effects and future challenges. Aquaculture 356-357, 351–356. doi: 10.1016/j.aquaculture.2012.04.046

[ref10] Daims H. Lebedeva E. V. Pjevac P. Han P. Herbold C. Albertsen M. . (2015). Complete nitrification by Nitrospira bacteria. Nature 528, 504–509. doi: 10.1038/nature16461, 26610024 PMC5152751

[ref11] Dang H. Chen R. Wang L. Shao S. Dai L. Ye Y. . (2011). Molecular characterization of putative biocorroding microbiota with a novel niche detection of epsilon- and Zetaproteobacteria in Pacific Ocean coastal seawaters. Environ. Microbiol. 13, 3059–3074. doi: 10.1111/j.1462-2920.2011.02583.x, 21951343

[ref12] Dang H. Lovell C. R. (2016). Microbial surface colonization and biofilm development in marine environments. Microbiol. Mol. Biol. Rev. 80, 91–138. doi: 10.1128/mmbr.00037-15, 26700108 PMC4711185

[ref13] Dong P. Guo H. Huang L. Zhang D. Wang K. (2023). Glucose addition improves the culture performance of Pacific white shrimp by regulating the assembly of Rhodobacteraceae taxa in gut bacterial community. Aquaculture 567:739254. doi: 10.1016/j.aquaculture.2023.739254

[ref14] Emerenciano M. G. C. Gaxiola G. Cuzon G. (2013). Biofloc technology (BFT): a review for aquaculture application and animal food industry. In Matovic M. D. (Ed.), Biomass Now - Cultivation and Utilization. IntechOpen. doi: 10.5772/53902 (Accessed December 31, 2025).

[ref15] Fierer N. Bradford M. A. Jackson R. B. (2007). Toward an ecological classification of soil bacteria. Ecology 88, 1354–1364. doi: 10.1890/05-1839, 17601128

[ref16] Fierer N. Lauber C. L. Ramirez K. S. Zaneveld J. Bradford M. A. Knight R. (2012). Comparative metagenomic, phylogenetic and physiological analyses of soil microbial communities across nitrogen gradients. ISME J. 6, 1007–1017. doi: 10.1038/ismej.2011.159, 22134642 PMC3329107

[ref17] Flegel T. W. (2019). A future vision for disease control in shrimp aquaculture. J. World Aquacult. Soc. 50, 249–266. doi: 10.1111/jwas.12589

[ref18] Franzosa E. A. Hsu T. Sirota-Madi A. Shafquat A. Abu-Ali G. Morgan X. C. . (2015). Sequencing and beyond: integrating molecular 'omics' for microbial community profiling. Nat. Rev. Microbiol. 13, 360–372. doi: 10.1038/nrmicro3451, 25915636 PMC4800835

[ref19] Guo H. Dong P. Gao F. Huang L. Wang S. Wang R. . (2022). Sucrose addition directionally enhances bacterial community convergence and network stability of the shrimp culture system. NPJ Biofilms Microbiomes 8:22. doi: 10.1038/s41522-022-00288-x, 35410335 PMC9001642

[ref20] Jespersen A. M. Christoffersen K. (1987). Measurements of chlorophyll-a from phytoplankton using ethanol as extraction solvent. Arch. Hydrobiol. 109, 445–454. doi: 10.1127/archiv-hydrobiol/109/1987/445

[ref21] Jiao Y. Zhao J. Sun N. Shi D. Xia D. Du Q. . (2025). The role of agricultural wastes—peanut shells in enhancing algae–bacteria consortia performance for efficient wastewater treatment. Water 17:485. doi: 10.3390/w17040485

[ref22] Jönsson L. J. Martín C. (2016). Pretreatment of lignocellulose: formation of inhibitory by-products and strategies for minimizing their effects. Bioresour. Technol. 199, 103–112. doi: 10.1016/j.biortech.2015.10.009, 26482946

[ref23] Khanjani M. H. Zahedi S. Sharifinia M. Hajirezaee S. Singh S. K. (2025). Biological removal of nitrogenous waste compounds in the biofloc aquaculture system – a review. Ann. Anim. Sci. 25, 3–21. doi: 10.2478/aoas-2024-0060

[ref24] Korenblum E. Jiménez D. J. van Elsas J. D. (2016). Succession of lignocellulolytic bacterial consortia bred anaerobically from lake sediment. Microb. Biotechnol. 9, 224–234. doi: 10.1111/1751-7915.12338, 26875750 PMC4767288

[ref25] Kuypers M. M. M. Marchant H. K. Kartal B. (2018). The microbial nitrogen-cycling network. Nat. Rev. Microbiol. 16, 263–276. doi: 10.1038/nrmicro.2018.9 29398704

[ref9002] Lan S. Guo X. Zhang D. Li X. Lv Z. Xie Y. (2023). Corn cob as a carbon source in ecological treatment of farmland runoff: Pollutant removal performance, effluent dissolved organic matter and microbial community structure. J. Water Process Eng. 56, 104525. doi: 10.1016/j.jwpe.2023.104525

[ref26] Lee S. J. Lee J. H. Yang X. Kim S. B. Lee J. H. Yoo H. Y. . (2015). Phenolic compounds: strong inhibitors derived from lignocellulosic hydrolysate for 2,3-butanediol production by *Enterobacter aerogenes*. Biotechnol. J. 10, 1920–1928. doi: 10.1002/biot.201500090, 26479290

[ref9008] Li P. Fu T. Cai A. Descovich K. Lian H. Gao T. , (2022). Effect of Peanut Shell and Rice Husk Bedding for Dairy Cows: An Analysis of Material Properties and Colostrum Microbiota. Animals, 12, 603. doi: 10.3390/ani12050603 35268172 PMC8909170

[ref27] Lin Y.-C. Chen J.-C. (2001). Acute toxicity of ammonia on *Litopenaeus vannamei* Boone juveniles at different salinity levels. J. Exp. Mar. Biol. Ecol. 259, 109–119. doi: 10.1016/S0022-0981(01)00227-1, 11325379

[ref28] Liu L. Tian C. Wang M. Luo Y. Huang Y. Jiang T. . (2025). Mutualism between degraders and nondegraders stabilizes the function of a natural biopolymer-degrading community. Proc. Natl. Acad. Sci. 122:e2500664122. doi: 10.1073/pnas.2500664122, 40690677 PMC12318217

[ref29] Louca S. Polz M. F. Mazel F. Albright M. B. N. Huber J. A. O’Connor M. I. . (2018). Function and functional redundancy in microbial systems. Nat. Ecol. Evol. 2, 936–943. doi: 10.1038/s41559-018-0519-1, 29662222

[ref30] Lulijwa R. Rupia E. J. Alfaro A. C. (2020). Antibiotic use in aquaculture, policies and regulation, health and environmental risks: a review of the top 15 major producers. Rev. Aquac. 12, 640–663. doi: 10.1111/raq.12344

[ref31] Lynd L. R. Weimer P. J. van Zyl W. H. Pretorius I. S. (2002). Microbial cellulose utilization: fundamentals and biotechnology. Microbiol. Mol. Biol. Rev. 66, 506–577. doi: 10.1128/mmbr.66.3.506-577.2002 12209002 PMC120791

[ref32] Manimekala T. Sivasubramanian R. Dar M. A. Dharmalingam G. (2025). Crafting the architecture of biomass-derived activated carbon via electrochemical insights for supercapacitors: a review. RSC Adv. 15, 2490–2522. doi: 10.1039/d4ra07682f, 39867323 PMC11758807

[ref33] Monlau F. Sambusiti C. Barakat A. Quéméneur M. Trably E. Steyer J. P. . (2014). Do furanic and phenolic compounds of lignocellulosic and algae biomass hydrolyzate inhibit anaerobic mixed cultures? A comprehensive review. Biotechnol. Adv. 32, 934–951. doi: 10.1016/j.biotechadv.2014.04.007, 24780154

[ref34] Papenfort K. Bassler B. L. (2016). Quorum sensing signal-response systems in gram-negative bacteria. Nat. Rev. Microbiol. 14, 576–588. doi: 10.1038/nrmicro.2016.89, 27510864 PMC5056591

[ref9006] Ray A. J. Leffler J. W. Browdy C. L. (2019). The effects of a conventional feed versus a fish-free feed and biofloc management on the nutritional and human sensory characteristics of shrimp (Litopenaeus vannamei). Aquac. Int. 27, 261–277. doi: 10.1007/s10499-018-0321-8

[ref35] Roller B. R. K. Stoddard S. F. Schmidt T. M. (2016). Exploiting rRNA operon copy number to investigate bacterial reproductive strategies. Nat. Microbiol. 1:16160. doi: 10.1038/nmicrobiol.2016.160, 27617693 PMC5061577

[ref36] Romano N. Zeng C. (2013). Toxic effects of ammonia, nitrite, and nitrate to decapod crustaceans: a review on factors influencing their toxicity, physiological consequences, and coping mechanisms. Rev. Fish. Sci. 21, 1–21. doi: 10.1080/10641262.2012.753404

[ref9004] Romano N. Dauda A. B. Ikhsan N. F. M. Karim M. M. A. Kamarudin M. S. (2018). Fermenting rice bran as a carbon source for biofloc technology improved the water quality, growth, feeding efficiencies, and biochemical composition of African catfish Clarias gariepinus juveniles. Aquac. Rep. 49, 3711–3720. doi: 10.1111/are.13837

[ref9007] Shi H. Zhao S. Wang K. Fan M. Han Y. Wang H. (2022). Effects of dietary astragalus membranaceus supplementation on growth performance, and intestinal morphology, microbiota and metabolism in common carp (cyprinus carpio). Aquac. Rep. 22, 100955. doi: 10.1016/j.aqrep.2021.100955

[ref37] Sicuro B. (2021). World aquaculture diversity: origins and perspectives. Rev. Aquac. 13, 1619–1634. doi: 10.1111/raq.12537

[ref38] Sloan W. T. Lunn M. Woodcock S. Head I. M. Nee S. Curtis T. P. (2006). Quantifying the roles of immigration and chance in shaping prokaryote community structure. Environ. Microbiol. 8, 732–740. doi: 10.1111/j.1462-2920.2005.00956.x, 16584484

[ref39] Tilman D. (2020). Resource Competition and Community Structure. Princeton University Press. Available online at: 10.1515/9780691209654 (Accessed December 31, 2025).

[ref40] Torres-Lagos E. Henríquez-Castillo C. Méndez C. Morales M. C. Cárcamo C. B. Navarrete P. . (2024). Biofloc culture system shapes the structure and function of environmental and intestinal bacterial communities in the river prawn *Cryphiops caementarius*. Aquac. Rep. 39:102359. doi: 10.1016/j.aqrep.2024.102359

[ref41] Wagner-Döbler I. Ballhausen B. Berger M. Brinkhoff T. Buchholz I. Bunk B. . (2010). The complete genome sequence of the algal symbiont *Dinoroseobacter shibae*: a hitchhiker's guide to life in the sea. ISME J. 4, 61–77. doi: 10.1038/ismej.2009.94, 19741735

[ref42] Wang Z. Tian H. Yang J. Shi H. Pan S. Yao Y. . (2020). Coupling of phosphorus processes with carbon and nitrogen cycles in the dynamic land ecosystem model: model structure, parameterization, and evaluation in tropical forests. J. Adv. Model. Earth Syst. 12:e2020MS002123. doi: 10.1029/2020MS002123

[ref9009] Wang Y. Xu L. Sun X. Wan X. Sun G. Jiang R. . (2020). Characteristics of the fecal microbiota of high- and low-yield hens and effects of fecal microbiota transplantation on egg production performance. Res. Vet. Sci. 129, 164–173. doi: 10.1016/j.rvsc.2020.01.020 32036124

[ref43] Wei Y. Liao S.-A. Wang A.-l. (2016). The effect of different carbon sources on the nutritional composition, microbial community and structure of bioflocs. Aquaculture. 465, 88–93. doi: 10.1016/j.aquaculture.2016.08.040

[ref44] Wu J. Chen Y. Xu X. Ren W. Zhang X. Cai X. . (2022). Screening of bioflocculant and cellulase-producing bacteria strains for biofloc culture systems with fiber-rich carbon source. Front. Microbiol. 13:969664. doi: 10.3389/fmicb.2022.969664 36504821 PMC9729547

[ref9001] Yadav N. K. Paul S. Patel A. B. Mahanand S. S. Biswas P. Choudhury T. G. . (2025). The role of biofloc technology in sustainable aquaculture: nutritional insights and system efficiency. Blue Biotechnology, 2, 7. doi: 10.1186/s44315-025-00023-4

[ref45] Yao X. W. Xu K. L. Yan F. Wang B. B. (2015). Thermogravimetric-mass spectrometry analysis and pyrolysis kinetic of peanut shell. J. Northeast. Univ. Nat. Sci. 36:1761. doi: 10.12068/j.issn.1005-3026.2015.12.020

[ref46] Zhang N. Zhou Y. Ali A. Wang T. Wang X. Sun X. (2024). Effect of molasses addition on the fermentation quality and microbial community during mixed microstorage of seed pumpkin peel residue and sunflower stalks. Fermentation 10:314. doi: 10.3390/fermentation10060314

