# Supplementary material for: Effects of peanut shells serving as a carbon source and microbial carrier on bacterial community structure in Penaeus vannamei culture water
Source: Front Microbiol. 2026 Apr 10;17:1778957. doi: 10.3389/fmicb.2026.1778957 (PMC13106462; doi:10.3389/fmicb.2026.1778957)
Supplement: Supplementary file 2 [file Supplementary_file_1.DOCX]

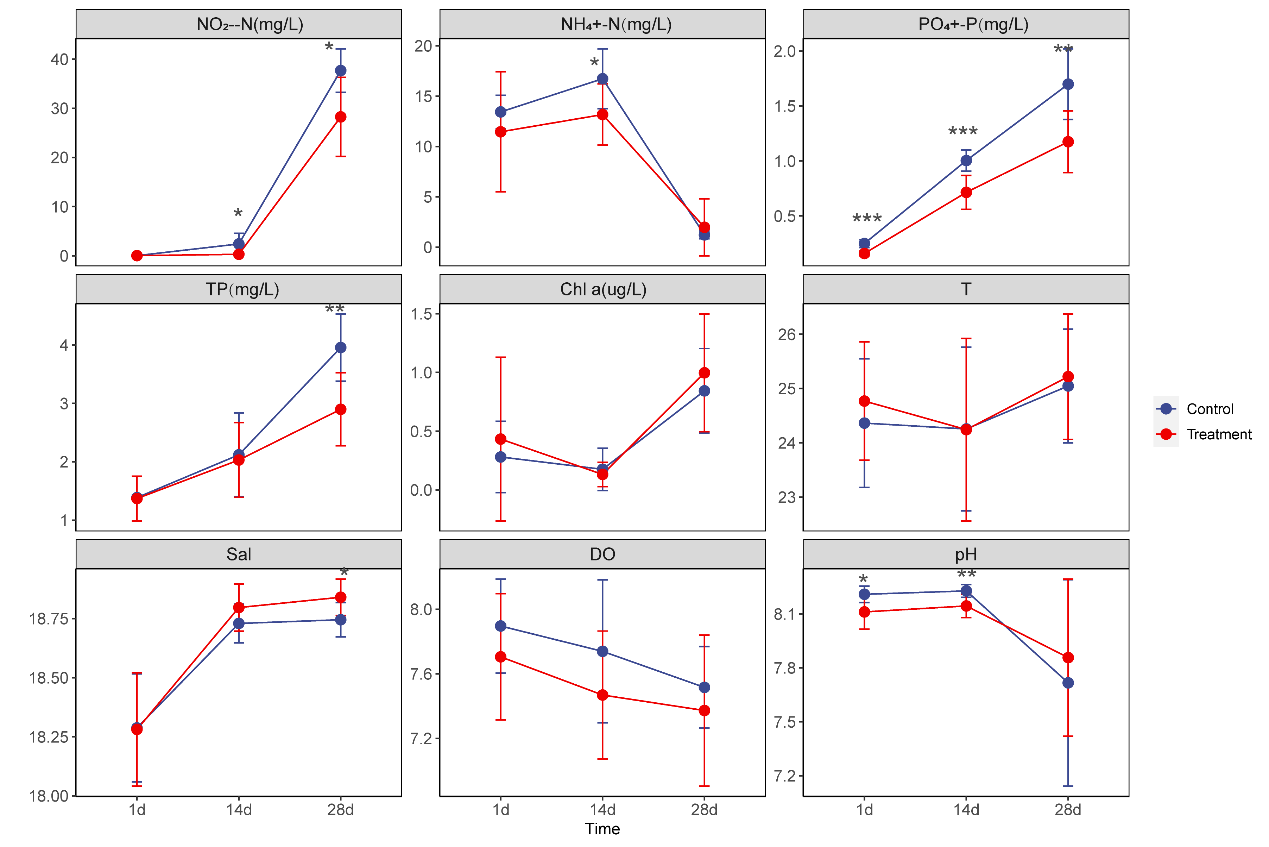


**Figure S1.** Dynamics of water quality parameters in control and peanut-shell-added groups during the 28-day culture period. Concentrations of ammonium (NH₄⁺-N), nitrite (NO₂⁻-N), and chlorophyll a (Chl a) were measured on days 1, 14, and 28. Data are presented as mean ± standard deviation (n=3). Following a significant interaction effect in a two-way ANOVA, independent-sample t-tests were conducted at each time point. Asterisks denote significant differences between control and treatment groups (**P* < 0.05, ***P* < 0.01, ****P* < 0.001).


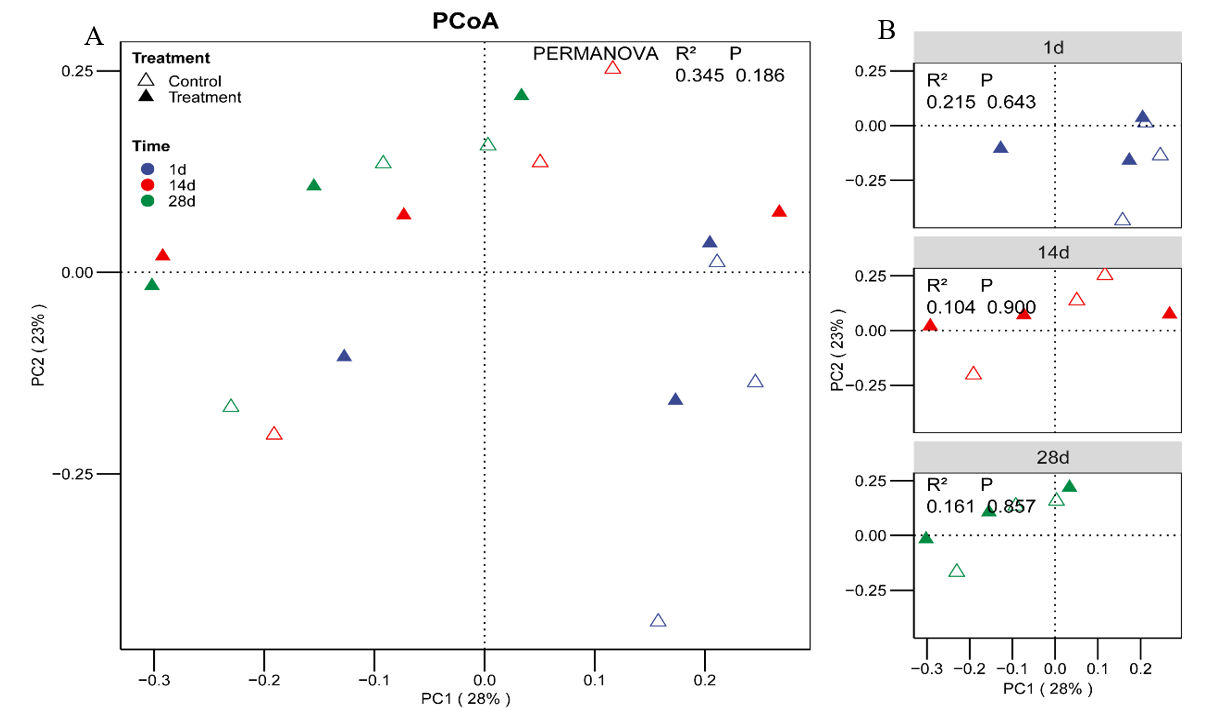


**Fig S2**. Bacterial β-diversity in rearing water of the control and peanut-shell-added groups.

(A) Principal coordinate analysis (PCoA) based on Bray–Curtis dissimilarity showing community separation across days 1, 14, and 28 (open triangles = control; solid triangles = peanut-shell group). PERMANOVA results: R² = 0.345, *P* = 0.186 (no significant group difference).

(B) Time-stratified PCoA illustrating bacterial community structure within each sampling day (1, 14, 28 d). No significant group difference was observed across time points (all PERMANOVA *P* > 0.05).

**Table S1**. Comparison of intestinal bacterial community composition in shrimp between the control and floc groups based on Bray-Curtis dissimilarity using Analysis of Similarities (ANOSIM) and Permutational Multivariate Analysis of Variance (PERMANOVA) (Permutations = 999).

|  | PERMANOVA | | ADONIS | |
| --- | --- | --- | --- | --- |
|  | Variation（R^2^） | P_adj_BH | R | P_adj_BH |
| 1d | 0.215 | 0.643 | 0.148 | 0.500 |
| 14d | 0.104 | 0.900 | -0.222 | 0.857 |
| 28d | 0.161 | 0.857 | -0.259 | 0.900 |

**Table S2.**Relative abundance of dominant bacterial phyla and proteobacterial classes in the shrimp intestine, presented as mean ± SD, and compared by one-way ANOVA.

|  | Control | Treatment | F | P |
| --- | --- | --- | --- | --- |
| 1d |  |  |  |  |
| Bacteroidetes | 0.160±0.022 | 0.248±0.058 | 6.082 | 0.069 |
| Actinobacteria | 0.381±0.202 | 0.256±0.005 | 1.149 | 0.344 |
| Deltaproteobacteria | 0.044±0.062 | 0.058±0.043 | 0.090 | 0.779 |
| Planctomycetes | 0.012±0.012 | 0.018±0.007 | 0.540 | 0.503 |
| Gammaproteobacteria | 0.069±0.082 | 0.028±0.003 | 0.748 | 0.436 |
| Chloroflexi | 0.013±0.009 | 0.004±0.003 | 2.543 | 0.186 |
| 14d |  |  |  |  |
| Bacteroidetes | 0.305±0.058 | 0.263±0.136 | 0.250 | 0.643 |
| Actinobacteria | 0.158±0.091 | 0.246±0.064 | 1.872 | 0.243 |
| Deltaproteobacteria | 0.043±0.020 | 0.032±0.043 | 0.174 | 0.698 |
| Planctomycetes | 0.038±0.013 | 0.018±0.008 | 4.807 | 0.093 |
| Gammaproteobacteria | 0.002±0.002 | 0.001±0.001 | 0.702 | 0.449 |
| Chloroflexi | 0.023±0.014 | 0.007±0.008 | 2.769 | 0.171 |
| 28d |  |  |  |  |
| Bacteroidetes | 0.269±0.062 | 0.317±0.095 | 0.526 | 0.509 |
| Actinobacteria | 0.164±0.073 | 0.159±0.025 | 0.013 | 0.914 |
| Deltaproteobacteria | 0.007±0.004 | 0.022±0.015 | 2.839 | 0.167 |
| Planctomycetes | 0.047±0.028 | 0.034±0.025 | 0.346 | 0.588 |
| Gammaproteobacteria | 0.006±0.002 | 0.005±0.001 | 1.451 | 0.295 |
| Chloroflexi | 0.044±0.047 | 0.014±0.009 | 1.178 | 0.339 |

**Table S3**. Relative abundance of dominant bacterial families in the shrimp aquaculture water environment, presented as mean ± SD, and compared by one-way ANOVA.

|  | Control | Treatment | F | P |
| --- | --- | --- | --- | --- |
| 1d |  |  |  |  |
| Rhodobacteraceae | 0.244±0.149 | 0.304±0.009 | 0.471 | 0.530 |
| Flavobacteriaceae | 0.142±0.009 | 0.155±0.039 | 0.307 | 0.609 |
| Microbacteriaceae | 0.350±0.204 | 0.163±0.017 | 2.497 | 0.189 |
| Saprospiraceae | 0.015±0.012 | 0.092±0.061 | 4.525 | 0.101 |
| Cellulomonadaceae | 0.015±0.010 | 0.041±0.032 | 1.857 | 0.245 |
| Phyllobacteriaceae | 0.036±0.014 | 0.044±0.035 | 0.120 | 0.747 |
| Pirellulaceae | 0.010±0.011 | 0.014±0.005 | 0.391 | 0.566 |
| Nannocystaceae | 0.039±0.063 | 0.031±0.009 | 0.040 | 0.850 |
| Propionibacteriaceae | 0.009±0.007 | 0.022±0.008 | 4.685 | 0.096 |
| Caldilineaceae | 0.011±0.010 | 0.004±0.003 | 1.852 | 0.245 |
| 14d |  |  |  |  |
| Rhodobacteraceae | 0.382±0.097 | 0.396±0.090 | 0.033 | 0.864 |
| Flavobacteriaceae | 0.268±0.056 | 0.183±0.048 | 4.071 | 0.114 |
| Microbacteriaceae | 0.102±0.050 | 0.117±0.086 | 0.074 | 0.799 |
| Saprospiraceae | 0.035±0.027 | 0.080±0.098 | 0.592 | 0.485 |
| Cellulomonadaceae | 0.037±0.039 | 0.065±0.041 | 0.743 | 0.437 |
| Phyllobacteriaceae | 0.035±0.017 | 0.028±0.010 | 0.353 | 0.584 |
| Pirellulaceae | 0.035±0.012 | 0.017±0.007 | 4.963 | 0.090 |
| Nannocystaceae | 0.036±0.030 | 0.027±0.043 | 0.075 | 0.798 |
| Propionibacteriaceae | 0.011±0.003 | 0.036±0.027 | 2.452 | 0.192 |
| Caldilineaceae | 0.021±0.014 | 0.007±0.008 | 2.258 | 0.207 |
| 24d |  |  |  |  |
| Rhodobacteraceae | 0.430±0.069 | 0.419±0.080 | 0.033 | 0.864 |
| Flavobacteriaceae | 0.238±0.054 | 0.221±0.020 | 0.253 | 0.642 |
| Microbacteriaceae | 0.117±0.044 | 0.064±0.015 | 3.924 | 0.119 |
| Saprospiraceae | 0.025±0.015 | 0.093±0.098 | 1.411 | 0.301 |
| Cellulomonadaceae | 0.026±0.028 | 0.044±0.026 | 0.656 | 0.463 |
| Phyllobacteriaceae | 0.018±0.010 | 0.017±0.003 | 0.031 | 0.868 |
| Pirellulaceae | 0.037±0.021 | 0.028±0.021 | 0.323 | 0.600 |
| Nannocystaceae | 0.004±0.003 | 0.002±0.002 | 0.984 | 0.377 |
| Propionibacteriaceae | 0.012±0.010 | 0.039±0.026 | 2.857 | 0.166 |
| Caldilineaceae | 0.042±0.046 | 0.013±0.010 | 1.109 | 0.352 |
